# Genome-wide association analyses for boar taint components and testicular traits revealed regions having pleiotropic effects

**DOI:** 10.1186/s12863-015-0194-z

**Published:** 2015-04-09

**Authors:** Christine Große-Brinkhaus, Leonie C Storck, Luc Frieden, Christiane Neuhoff, Karl Schellander, Christian Looft, Ernst Tholen

**Affiliations:** Institute of Animal Science, University of Bonn, Endenicher Allee 15, 53115 Bonn, Germany

**Keywords:** Boar taint, Androstenone, Testis size, GWAS, Pleiotropy, Pigs

## Abstract

**Background:**

The aim of this study was to perform a genome-wide association analyses (GWAS) for androstenone, skatole and indole in different Pietrain sire lines and compare the results with previous findings in purebred populations. Furthermore, the genetic relationship of androstenone and skatole were investigated with respect to pleiotropy. In order to characterize the performance of intact boars, crossbred progenies of 136 Pietrain boars mated to crossbred sows from three different breeding companies were tested on four test stations. A total of 598 boars were performance tested according to the rules of stationary performance testing in Germany. Beside common fattening and carcass composition traits, the concentrations of the boar taint components and testicular size parameters were recorded. All boars were genotyped with the PorcineSNP60 Illumina BeadChip. The GWAS were performed using the whole data set as well as in sub groups according to the line of origin. Besides an univariate GWAS approach, principal component (PC) techniques were applied to identify common expression pattern affecting the biosynthesis and the metabolism of androstenone.

**Results:**

In total, 33 SNPs were significantly associated with at least one of the boar taint components. Only one SNP was identified being significant in both subgroups. The analyses of the testes size parameters revealed 31 significant associations. The numbers of significant SNPs within the genetic groups evidenced the strong population specific effects. A multivariate approach using PC revealed 33 significant associations for five different PC.

**Conclusions:**

Based on Pietrain sired cross bred boars, the mayor objective of our study was to identify QTL for boar taint components and to detect pleiotropy among boar taint and testes traits. The high number of identified QTL revealed that boar taint traits are influenced by a large number of loci. Analyzing pleiotropy allowed identifying a QTL affecting androstenone and the gonasomatic index. In this region, QTL for ovulation rate and age at puberty of sows have been described in literature. This supports the physiological findings that the androstenone level of boars and reproduction performance of sows might be linked by an antagonistic relationship.

**Electronic supplementary material:**

The online version of this article (doi:10.1186/s12863-015-0194-z) contains supplementary material, which is available to authorized users.

## Background

Economically relevant proportions of meat from intact boars, ranging from 5 to 40%, can be characterized by an unpleasant odor and an accompanying taste, known as boar taint [1]. In order to avoid the development of boar taint, piglets are castrated without anesthesia which is painful and unacceptable because of animal welfare reasons. Within the European Union, the surgical castration without anesthesia will be banned as of 2018 [2]. Alternatives like surgical castration with anesthesia, immune castration and boar fattening are controversially discussed. Boar fattening has several advantages including higher feed efficiency and better carcass values. However, the risk of tainted meat has to be controlled and the percentages of tainted carcasses have to be reduced. Beside adapted housing systems and feeding regimes, reduction of tainted boars can be achieved by selection strategies within breeding programs [3,4].

Boar taint is mainly caused by the accumulation of androstenone and skatole in fat tissue. Androstenone (5α-androst-16-en-3-one) is produced by the Leydig cells of the testis along with other sexual steroids including testosterone and estrogens, but leads to a urine-like odour (reviewed in [5,6]). Skatole (3-methylindole) as well as indole are derivatives of tryptophan metabolism and are synthesized by intestinal bacteria in the hindgut of pigs [7,8], but only skatole is correlated with a strong fecal odour [9]. Androstenone is degraded in liver and salivary gland, whereas both boar taint components, androstenone and skatole, are mainly degraded in liver and share some common enzyme families involved in the metabolism [10].

Heritability (h^2^) and genetic correlations for androstenone and skatole levels were investigated in different breeds by several researchers (e.g. [3,11-14]). The h^2^ for androstenone ranges from 0.5 to 0.7 and for skatole from 0.3 to 0.5 in different pig breeds and crosses. The levels of skatole are influenced by environmental factors like feeding, husbandry and hygiene management that affect the bacterial metabolism and therefore lead to a lower heritability compared to androstenone. Genetic relationships between androstenone and skatole were estimated between 0.3 and 0.4. Much higher genetic correlations were reported for skatole and indole (r_g_ = 0.71 to 0.78), because both traits are associated with the tryptophan metabolism.

Quantitative trait loci (QTL) and genome-wide association studies (GWAS) were performed in order to clarify the genetic basis of boar taint [15-17]. In these studies pure bred populations including Large White, Landrace and Duroc, experimental resource population or populations of commercial sire line pigs were investigated. Until now more than 90 QTL for boar taint components or pork odor have been identified on 15 autosomes ([PigQTLdb, http://www.animalgenome.org/cgi-bin/QTLdb/SS/index, [18]). In general, there is a limited number of QTL with a major impact on boar taint traits. In order to avoid antagonistic pleiotropy, particular on fertility traits, it is useful to clarify the biological background of these QTL. However, there are serious indications, that breeding against androstenone might affect reproductive traits negatively [19,20].

Regarding paternal reproduction performance, Baes *et al.* [21] detected small negative effects between androstenone and semen quality and quantity. Phenotypic records for paternal reproduction performance often originated from preselected artificial insemination (AI) boars from AI stations. Boars with extreme negative fertility parameters are hardly investigated because these boars are culled before their use in AI stations.

Testicular morphology parameters are good predictors of paternal fertility performance as it has been shown in several studies [22-24]. As organs of spermatogenesis and secretion of androgen in mammals [25,26], testes weight and androstenone have been reported to be closely linked leading to moderate genetic correlations of 0.41-0.47 [27]. Moreover, a joint analysis of gonadosomatic index and androstenone might be helpful in order to characterize pleiotropic and possible antagonistic effects between boar taint and male fertility. In addition, the investigation of pleiotropy is promising to dissect the genetic relationships between traits [28].

In this study, GWAS for boar taint components (androstenone, skatole and indole) and testicular morphology (testes size, length, width and gonadosomatic index) in different Pietrain sire lines were performed to identify promising QTL regions. In Germany, crosses of hybrid sows and Pietrain boars are usually used in order to produce fattening pigs. In addition we aimed to investigate pleiotropy between boar taint components and paternal fertility traits. It has been shown by Frieden *et al.* [29] who estimated positive genetic correlations between boar taint traits and testes morphological parameters (see Additional file 1: Table S1a and S1b). For this, joint analyses based on principal component approaches were applied in order to verify the described physiological and genetical relationships between androstenone and testical morphological parameters.

## Methods

### Animal resources

The study is based on intact boars of type Pietrain × crossbred sow. In total 136 Pietrain AI-sires were mated with 410 different crossbred dams that originated from three different breeding organizations. The first two lines were F1 crosses of German Edelschwein and German Landrace of two breeding organizations (cross I and II), while the third line was a F2 sow based on Large White × (Leicoma × German Landrace) (cross III). Out of 1010 pigs, 603 boars were selected for genotyping. All population pedigrees included families with 1 to 2 offsprings in a half-sib family structure and in total 243 intact boars belonged to cross I, 238 to cross II and 212 to cross III. For detailed information about the population please see Frieden *et al.* [1,29]. Pigs were fattened under standardized conditions on five different performance testing stations located in North Rhine-Westphalia, Baden-Württemberg and Bavaria. Newly born boars were raised on farm to a weight of 25 kg and were brought to the performance testing stations where the animals were kept in single and group pens with 12 to 14 boars. All animals were fed ad libitum with uniform standard diet that was used in stationary sib and progeny testing in Germany. Additional information about the test station can be found in the regulation for pig performance testing [30]. The boars were slaughtered from November 2009 until December 2010.

Data recording and sample collection were conducted strictly in line with the German law on animal welfare. The entire experiment including applied standard operating procedures was approved by the veterinary and food inspection, Siegburg, Germany (No. 39600305-547/15).

### Trait analysis

The pigs were slaughtered in commercial abattoirs or in research abattoirs directly connected to the testing stations. Slaughterhouse management gave the necessary permissions for the tissue and organ collection. The samples of muscle, testis and fat were afterwards stored at −20°C. Growth, carcass and meat quality traits were collected according to guidelines of the German stationary performance test. In addition, both testicles were collected immediately after slaughtering. Length, width and weight without scrotum and epididymis were recorded for each testicle. Gonadosomatic index (GSI, %) was calculated dividing testis mass by body weight. The GSI allows to characterize the sexual maturity of animals related to testes development and sperm production [31].

The quantitative determination of the three main components of boar taint was conducted by Frauenhofer Institute for Molecular Biology and Applied Ecology (IME) Schmallenberg. In back fat androstenone was evaluated using gas chromatography/mass spectrometry (GC/MS) [32]. The content of skatole and indole in backfat was purified by reverse-phase high performance liquid chromatography (RP-HPLC) [33]. The values of androstenone, skatole and indole were log-transformed using log base *e* in order to meet the assumption of Gaussian distributed data.

### Genotyping and data validation

Genomic DNA was isolated from muscle samples using the BTS-kit (Blood, Tissue, Semen, CMG-1046) from Chemagen (PerkinElmer Chemagen Technologie GmbH, Baesweiler, Germany). Single nucleotide polymorphisms (SNPs) of all boars were genotyped on the PorcineSNP60 [34] Illumina iSelect BeadChip according to the protocol [35]. For quality control, data were analyzed by the R-package GenABEL to account for low call rate (CALL > 0.95), low minor allele frequency (MAF > 0.01) and deviation from Hardy-Weinberg equilibrium (p-value < 0.001) [36]. After quality control five samples were removed from the data set because of low call rate, so that 598 samples with 43,527 SNPs were left for further analysis.

The SNPs were annotated by the Pig Sscrofa10.2 (International Swine Genome Sequencing Consortium) [37].

### Population structure

GWAS was performed within the whole population and in clusters comprising different subpopulations. In order to visualize possible population stratification the first two and three principal components of the genomic based kinship matrix G were visualized in a plot.

The kinship coefficients G from genomic data were estimated using the formula [38]:

1 $$ {\mathrm{G}}_{\mathrm{ij}}=\frac{1}{\mathrm{n}}{\displaystyle \sum_{\mathrm{k} = 1}^{\mathrm{n}}}\frac{\left({\mathrm{g}}_{\mathrm{ik}}-{\mathrm{p}}_{\mathrm{k}}\right)\left({\mathrm{g}}_{\mathrm{ik}}-{\mathrm{p}}_{\mathrm{k}}\right)}{{\mathrm{p}}_{\mathrm{k}}\left(1-{\mathrm{p}}_{\mathrm{k}}\right)} $$

where g_ik_ is the genotype of the i^th^ individual at the k^th^ SNP, p_k_ is the frequency of the major allele and n is the number of SNPs used for kinship estimation.

Based on the visualized genetic distances, the population was separated into two sub-populations. In addition, quality control was performed for the genetic data of the defined sub-population as well.

### Genome-wide association analysis

The GWAS was performed with the genome-wide rapid association using mixed model and regression (GRAMMAR) [39,40]. GRAMMAR uses a score test to identify associations between SNP genotypes and traits residuals. In general this approach can be separated into two steps: (1) Pre-correction of the phenotype and (2) the association test. Trait residuals were calculated for all traits by means of a polygenic model containing the genomic-based kinship matrix:

2 $$ {y}_{\mathrm{ijkl}} = \mu +{S}_j+{H}_k+{\left(\mathrm{L}\times \mathrm{C}\right)}_l + {\beta}_1{\mathrm{weight}}_i + {\beta}_2{\mathrm{age}}_i + {a}_i + {e}_i $$

where y_i_ is the phenotype of the i^th^ individual. As fixed effects season (S), husbandry (H), performance testing station (L) nested with type of cross (C) and as covariates slaughter weight (weight) and slaughter age (age) with regression coefficients β are implemented in the model. a_i_ ~ N(0,Gσ^2^_a_) are the random additive polygenic effects and e_i_ are the random residual effects. The kinship coefficients were estimated following formula (1). The influencing effects were considered according to the study by Frieden *et al.* [29].

The trait residuals were estimated as

3 $$ {\mathrm{y}}_{\mathrm{i}\mathrm{jkl}}^{*}=\mathrm{y}-\left(\hat{\upmu}+{S}_j+{H}_k+{\left(\mathrm{L}\times \mathrm{C}\right)}_l + {\beta}_1{\mathrm{weight}}_i + {\beta}_2{\mathrm{a}\mathrm{ge}}_i+\hat{{\mathrm{a}}_{\mathrm{i}}}\right) $$

In a second step, the test for association was performed with these trait residuals using the following linear model:

4 $$ {\mathrm{y}}_{\mathrm{i}}^{*}={\upmu}^{*}+{\mathrm{kg}}_{\mathrm{i}}+{\mathrm{e}}_{\mathrm{i}}^{*} $$

where y* represents vector of i^th^ observations (residuals from (3)), μ* the intercept, k is the regression on the genotype (g_i_), where g contains a dose effect of a target allele for each SNP and e* is the random residual [39]. A χ^2^ test-statistic is used to determine whether the SNP is significant associated with the trait.

In order to verify remaining population stratification, the inflation factor λ, which depends on the squared original test statistic of the i^th^ SNP ${T}_i^2$ was calculated as

5 $$ \lambda =\frac{Median\ \left({T}_i^2\right)}{0.4549} $$

The inflation factor λ and the observed versus the expected p-values for each SNP are illustrated in quantile-quantile (Q-Q) plots for each trait.

In order to correct for multiple testing, chromosome-wide Bonferroni significance levels (p < 0.05) were calculated from the number of SNPs representing the different chromosomes. The genome-wide critical values for the significance levels of the empirical p-value associated with type I errors where α = 0.05 and α = 0.01 were 1.18E-06 and 2.37E-07, respectively. All chromosome-wide critical values of the empirical p-value are given in Additional file 1: Table S2.

The variance explained by the respective SNP was calculated using following formula:

6 $$ \mathrm{V}\mathrm{a}\mathrm{r}\ \left(\%\right) = \frac{\chi_{1df}^2}{\left(n-2+{\chi}_{1df}^2\right)}\times 100 $$

where χ^2^ is the result of the score test as implemented in GenABEL package and n the number of individuals. This formula resulted from the transformation of a Student’s t-distribution into a z-distribution [41].

Identified regions were further analyzed calculating pairwise linkage disequilibrium (LD) between SNPs and determine haplotype blocks. This allows to display the relationship between associated and non-associated surrounding SNPs of a particular region. LD was measured as r^2^ between SNPs using Haploview (version 4.2 [42]).

### Analysis of pleiotropy

In order to detect pleiotropic QTLs for boar taint and paternal fertility traits, a principal component analysis (PCA) was performed. Principal components analysis, a widely used data reduction method, explains the variance–covariance structure in terms of uncorrelated linear combinations of the original variables.

Weller *et al.* [43] proposed to use PCA for multitrait detection of pleiotropic QTL. Gilbert and le Roy [44] showed that multitrait QTL detection using PCA on phenotypes increased the detection power and accuracy. The PCA is performed from the phenotypic covariance matrix of the data, considered as estimations of the residual covariance matrix. Analyzing *p* traits result in *p* phenotypically uncorrelated linear combinations derived from the components of the eigenvectors of the phenotypic covariance matrix. Each eigenvalue represents the part of phenotypic variability explained by the associated principal component variable [44]. Further detailed information can be found in Gilbert and le Roy [44].

In order to investigate the biosynthesis and metabolism of androstenone, two different PCAs were performed. In the first PCA, androstenone and gonadosomatic index were investigated jointly. In a second PCA, the three boar taint components androstenone, skatole and indole were analyzed.

The resulting principal components (PCs) were treated as separate traits and included in the analysis.

## Results

### Genotyping, population stratification and data validation

**Figure 1:**
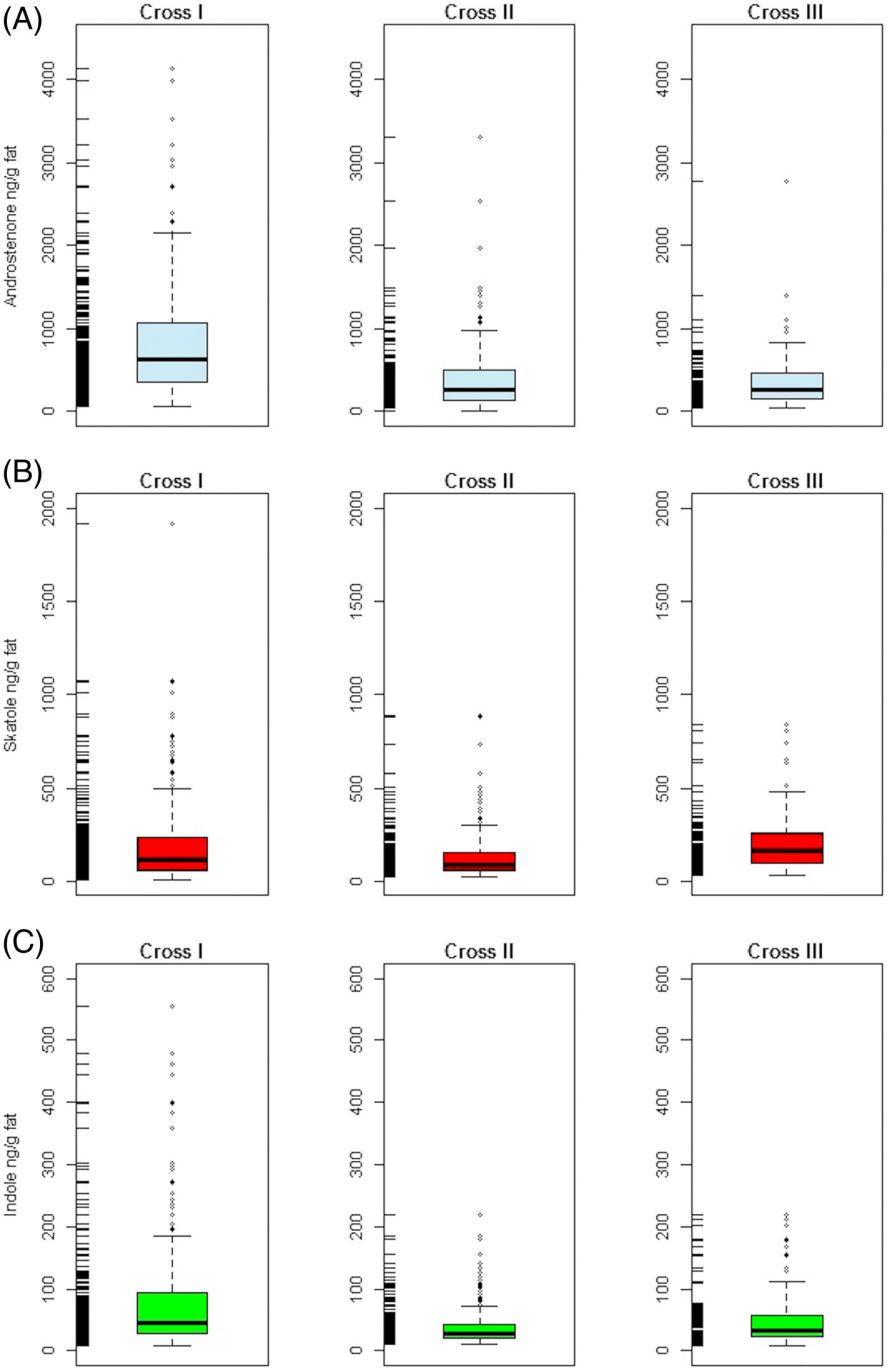
**Whiskers plots of the distribution of the untransformed concentrations of the three boar taint components for the three crosses.** Plot **(A)** - androstenone ng/g fat, plot **(B)** - skatole ng/g fat and plot **(C)** - indole ng/g fat.

The descriptive statistics of phenotypic measurements of the investigated boars is given in Table 1. Animals were slaughtered at a mean age of 175 (±14.6) days with an average hot carcass weight of 90.1 (±6.3) kg. The distributions of androstenone, skatole and indole separated by type of crosses are presented as box-and-whisker plots in Figure 1. These graphs showed distinct differences in the concentration of androstenone in fat between cross I compared to cross II and III. Similar results were observed for the traits skatole and indole. No significant differences between crosses were found for testicular traits.

**Table 1:** **Descriptive statistics for boar taint components and finishing traits**

**Trait**	**N**	**mean (± SD)**	**min**	**max**
Boar taint components				
Androstenone (ng/g)	603	568.50 (663.66)	3.50	6458.10
Skatole (ng/g)	603	169.11 (182.80)	7.80	1915.00
Indole (ng/g)	603	58.09 (66.52)	7.50	554.40
Testicular mean weight (g)^1^	586	226.04 (59.05)	61.70	376.00
Testicular mean length (mm)^1^	585	101.75 (14.62)	45.00	129.50
Testicular mean width (mm)^1^	585	63.77 (9.30)	29.00	84.50
Gonadosomatic index (%)^2^	556	4.06 (0.91)	1.13	6.54
Finishing traits				
Back fat thickness (cm)	603	1.69 (0.32)	0.40	2.60
Hot carcass weight (kg)	603	90.14 (6.30)	71.51	115.63
Age at slaughter (days)	602	174.70 (14.62)	144.00	219.00

Mean, standard deviation (SD), minimum (min), maximum (max) values for investigated traits, number of observations (N), ^1^mean weight, length and width of both testes, ^2^testes mass divided by body weight.

**Figure 2:**
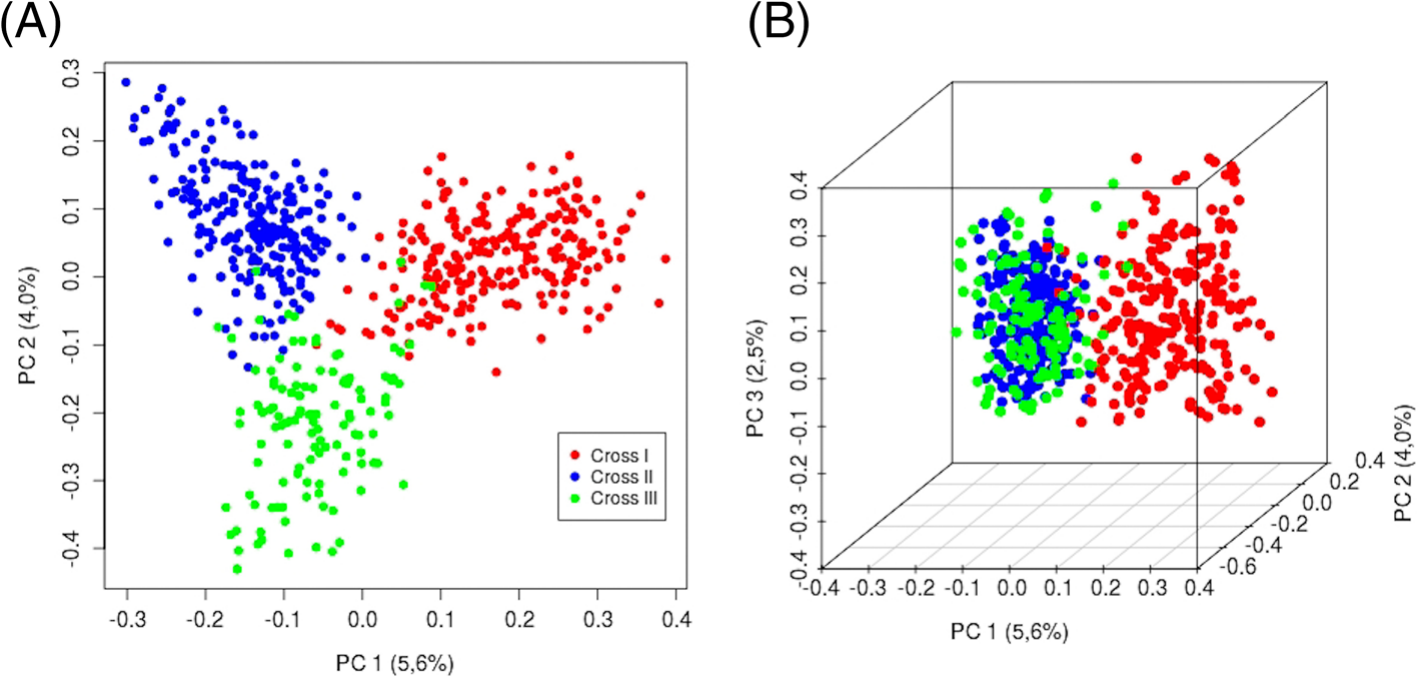
**Plots of the first two (A) and three (B) principal components showing the genomic kinship between the analyzed animals.** The plots visualize the overall genetic distances between the boars based on 43.527 SNP markers.

A total of 603 animals were genotyped on the Illumina porcineSNP60 BeadChip resulting in 43,527 segregating SNPs. Five samples had a call rate < 0.95 and were removed from the data set. We calculated genome-wide pairwise identity-by-state distances and quantified the population stratification (Figure 2). Based on the genetic distances of the individuals, as presented by the two-dimensional figure using principal component analysis, the animals were grouped in three subpopulations (Figure 2A). The investigation of a third principal component revealed, that the animals of cross II and cross III had a closer relationship compared with the boars of cross I (Figure 2B). In order to verify the consequences of population specific effects, we analyzed the entire data set (data set A) and the subset B1 and B2, which contained pigs from cross I (B1) or II and III (B2), respectively.

The quality control of the two subpopulations revealed for subset B1 242 individuals with 48,899 segregating markers and for subset B2 356 individuals and 48,409 segregating markers. Different numbers of segregating SNP markers were mainly due to significant deviations from Hardy-Weinberg equilibrium.

### Genome-wide association analysis

The GWAS using a Bonferroni corrected p-value ≤ 0.05 showed that 28 SNPs located on autosomes were significantly associated with one of the boar taint components (Table 2, 3 and 4). Five associated SNPs have not yet been map to the porcine genome. Furthermore, four identified SNPs revealed a low minor allele frequency ranging from 0.03 to 0.06 and the homozygote minor genotype was not present in the dataset.

**Table 2:** **Chromosome wide significant associations identified in the whole data set for boar taint and testes size parameters**

**Trait** ^**1**^	**SNP**	**SSC** ^**2**^	**Pos** ^**3**^	**Mut** ^**4**^	**MAF** ^**5**^	**eEff (se)** ^**6**^	**Chi2**	**Emp. P-value** ^**7**^	**Var** ^**8**^
lnSKA	ASGA0047791	10	46867278	G/A	0.45	−0.10 (0.03)	14.32	1.93E-05*	2.35%
lnSKA	ALGA0064494	12	7132733	G/A	0.23	0.13 (0.03)	14.84	1.36E-05*	2.43%
lnSKA	ASGA0077381	17	54986042	G/A	0.04	0.28 (0.07)	14.03	2.35E-05*	2.30%
lnIND	ALGA0056841	10	10002850	G/A	0.24	0.13 (0.03)	15.54	1.38E-05*	2.55%
lnIND	H3GA0031761	11	31230971	A/G	0.43	−0.11 (0.03)	15.72	1.24E-05*	2.58%
lnIND	M1GA0020074	14	152454616	A/C	0.26	0.16 (0.03)	26.94	1.05E-08***	4.33%
lnIND	MARC0028756	14	152480709	A/G	0.26	0.16 (0.03)	26.94	1.05E-08***	4.33%
lnIND	SIRI0000194	14	153477507	A/G	0.32	0.15 (0.03)	24.63	4.48E-08***	3.97%
lnIND	ASGA0068311	14	153593360	A/G	0.32	0.15 (0.03)	24.70	4.29E-08***	3.99%
lnIND	MARC0059044	NA	NA	A/G	0.28	0.13 (0.03)	18.92	1.62E-06#	3.08%
length	ASGA0018288	4	11933017	A/G	0.24	−2.99 (0.73)	16.62	1.09E-05*	2.80%
length	H3GA0016069	5	23313949	A/G	0.32	−2.82 (0.66)	18.00	4.74E-06**	3.03%
length	ASGA0025083	5	23646360	A/G	0.33	−2.54 (0.65)	15.42	2.28E-05*	2.60%
length	H3GA0044665	15	91511319	G/A	0.43	−2.59 (0.66)	15.29	2.46E-05*	2.58%
length	CASI0001308	18	60484331	G/A	0.03	−7.17 (1.78)	16.23	1.39E-05*	2.74%
length	MARC0003381	NA	NA	A/G	0.43	−2.62 (0.60)	19.22	2.26E-06	3.22%
width	ALGA0097186	18	16444813	A/G	0.45	1.52 (0.39)	15.05	2.45E-05*	2.54%
width	ALGA0098863	18	57260307	A/G	0.35	−1.63 (0.42)	15.03	2.48E-05*	2.54%
weight	ALGA0031253	5	23002609	A/G	0.33	−7.19 (1.70)	17.88	1.29E-06**	3.00%
weight	ASGA0025080	5	23190382	A/C	0.40	−6.81 (1.71)	15.76	5.49E-06**	2.65%
weight	H3GA0016069	5	23313949	A/G	0.32	−7.62 (1.75)	19.05	5.84E-07***	3.20%
weight	ASGA0025083	5	23646360	A/G	0.33	−8.20 (1.70)	23.36	3.14E-08***	3.88%
weight	ALGA0031261	5	23662089	G/A	0.10	−10.12 (2.76)	13.45	2.67E-05*	2.27%
weight	H3GA0016074	5	23737420	A/G	0.24	−7.41 (1.94)	14.61	1.21E-05*	2.47%
weight	ASGA0103650	5	24057900	G/A	0.24	−7.44 (1.94)	14.73	1.12E-05*	2.48%
weight	ALGA0098863	18	57260307	A/G	0.35	−6.65 (1.80)	13.61	2.40E-05*	2.30%
weight	MARC0003381	NA	NA	A/G	0.44	−7.70 (1.57)	23.98	2.06E-08#	7.50%
GSI	ALGA0031253	5	23002609	A/G	0.33	−0.0089 (0.0021)	18.52	4.51E-07***	3.26%
GSI	H3GA0016069	5	23313949	A/G	0.32	−0.0085 (0.0021)	16.12	2.50E-06**	2.86%
GSI	ASGA0025083	5	23646360	A/G	0.33	−0.0096 (0.0021)	21.38	5.90E-08***	3.75%
GSI	H3GA0016074	5	23737420	A/G	0.24	−0.0088 (0.0024)	13.94	1.19E-05*	2.48%
GSI	ASGA0103650	5	24057900	G/A	0.24	−0.0089 (0.0024)	14.20	9.95E-06*	2.52%

^1^: androstenone, log-transformed (lnAND); skatole, log-transformed (lnSKA); indole, log-transformed (lnIND); testis length, width and weight (length, width and weight); gonadosomatic index (GSI); ^2^: *sus scrofa* chromosome (SSC); ^3^: position in Mb; ^4^: mutation (Mut); ^5^: minor allele frequency (MAF); ^6^: substitution effect and standard error (se), ^7^: empirical p-value and significant thresholds, Bonferroni corrected - ***p< 0.05 genome-wide,**p<0.01 chromosome-wide and *p<0.05 chromosome-wide significance level, #p<0.05 significance level of not annotated SNPs; ^8^: proportion of the explained variation (Var, %).

**Table 3:** **Chromosome wide significant associations identified in subset B1 for boar taint and testes size parameters**

**Trait** ^**1**^	**SNP**	**SSC** ^**2**^	**Pos** ^**3**^	**Mut** ^**4**^	**MAF** ^**5**^	**eEff (se)** ^**6**^	**Chi2**	**Emp. P-value** ^**7**^	**Var** ^**8**^
lnAND	ALGA0039432	7	23184219	G/A	0.48	0.13 (0.03)	14.73	1.27E-05*	5.80%
lnAND	MARC0059955	7	24223914	G/A	0.37	0.13 (0.03)	14.40	1.59E-05*	5.68%
lnAND	MARC0097446	18	3346636	C/A	0.03	0.36 (0.10)	13.55	2.83E-05*	5.36%
lnAND	ASGA0104833	NA	NA	G/A	0.23	0.15 (0.04)	15.61	7.01E-06#	6.13%
lnAND	ASGA0094873	NA	NA	A/G	0.48	−0.12 (0.03)	15.29	8.64E-06#	6.02%
lnSKA	H3GA0000454	1	6764028	A/C	0.45	−0.20 (0.05)	18.66	1.45E-06**	7.24%
lnSKA	ASGA0048539	10	63586433	G/A	0.13	0.24 (0.06)	14.05	2.91E-05*	5.55%
lnSKA	ASGA0077381	17	54986042	G/A	0.06	0.37 (0.09)	16.10	7.61E-06**	6.31%
lnIND	H3GA0000454	1	6764028	A/C	0.45	−0.22 (0.05)	17.27	5.55E-06*	6.74%
lnIND	H3GA0012922	4	77217709	G/A	0.36	−0.20 (0.05)	16.44	9.30E-06*	6.44%
lnIND	ALGA0025735	4	77249057	G/C	0.36	−0.20 (0.05)	16.44	9.30E-06*	6.44%
width	ASGA0098931	18	56634280	A/G	0.02	−12.76 (2.95)	18.74	7.88E-06**	7.75%
GSI	ALGA0108818	12	49085493	G/A	0.13	0.0003 (0.0001)	15.83	2.04E-05*	7.54%

^1^: androstenone, log-transformed (lnAND); skatole, log-transformed (lnSKA); indole, log-transformed (lnIND); testis length, width and weight (length, width and weight); gonadosomatic index (GSI); ^2^: *sus scrofa* chromosome (SSC); ^3^: position in Mb; ^4^: mutation (Mut); ^5^: minor allele frequency (MAF); ^6^: substitution effect and standard error (se), ^7^: empirical p-value and significant thresholds, Bonferroni corrected **p<0.01 chromosome-wide and *p<0.05 chromosome-wide significance level, #p<0.05 significance level of not annotated SNPs; ^8^: proportion of the explained variation (Var, %).

**Table 4:** **Chromosome wide significant associations identified in subset B2 for boar taint and testes size parameters**

**Trait** ^**1**^	**SNP**	**SSC** ^**2**^	**Pos** ^**3**^	**Mut** ^**4**^	**MAF** ^**5**^	**eEff (se)** ^**6**^	**Chi2**	**Emp. P-value** ^**7**^	**Var** ^**8**^
lnAND	M1GA0008473	6	30900258	C/A	0.18	−0.11 (0.03)	13.61	1.78E-05*	3.71%
lnAND	ALGA0106517	NA	NA	A/G	0.43	0.09 (0.02)	15.27	5.50E-06#	4.14%
lnSKA	DIAS0002096	7	22743118	C/A	0.03	0.30 (0.08)	15.12	1.19E-05*	4.10%
lnSKA	M1GA0014109	10	49859018	G/A	0.52	0.12 (0.03)	18.79	1.04E-06***	5.05%
lnSKA	ASGA0074339	16	78560312	A/G	0.27	0.11 (0.03)	13.72	3.01E-05*	3.73%
lnSKA	ASGA0098123	NA	NA	G/A	0.51	0.12 (0.03)	18.74	1.08E-06#	5.04%
lnIND	ASGA0068236	14	151808771	G/A	0.16	0.10 (0.02)	15.72	5.43E-06*	4.25%
lnIND	M1GA0020074	14	152454616	A/C	0.23	0.09 (0.02)	17.84	1.27E-06**	4.80%
lnIND	MARC0028756	14	152480709	A/G	0.23	0.09 (0.02)	17.84	1.27E-06**	4.80%
lnIND	MARC0102391	14	153374972	A/G	0.29	0.08 (0.02)	15.43	6.64E-06**	4.37%
lnIND	SIRI0000194	14	153477507	A/G	0.30	0.08 (0.02)	16.02	4.44E-06**	4.33%
lnIND	ASGA0068311	14	153593360	A/G	0.30	0.08 (0.02)	16.02	4.44E-06**	4.33%
width	ALGA0060242	11	1386376	A/G	0.07	1.03 (0.28)	13.81	1.33E-05**	3.78%
length	MARC0003381	NA	NA	A/G	0.42	−0.78 (0.18)	17.38	8.64E-07#	4.71%
weight	M1GA0015162	11	66901762	A/G	0.50	0.0026 (0.006)	10.44	2.34E-05*	2.88%
weight	MARC0003381	NA	NA	A/G	0.42	−0.0022 (0.006)	12.85	2.68E-06#	3.51%
GSI	ASGA0025083	5	23646360	A/G	0.31	−4.68E-05 (1.31E-05)	12.84	1.57E-05*	3.51%
GSI	ALGA0113147	15	171405	A/G	0.15	6.22E-05 (1.71E-05)	13.17	1.22E-05*	3.60%
GSI	MARC0003381	NA	NA	A/G	0.42	−5.00E-05 (1.20E-05)	17.34	5.20E-07#	4.68%

^1^: androstenone, log-transformed (lnAND); skatole, log-transformed (lnSKA); indole, log-transformed (lnIND); testis length, width and weight (length, width and weight); gonadosomatic index (GSI); ^2^: *sus scrofa* chromosome (SSC); ^3^: position in Mb; ^4^: mutation (Mut); ^5^: minor allele frequency (MAF); ^6^: substitution effect and standard error (se), ^7^: empirical p-value and significant thresholds, Bonferroni corrected - ***p< 0.05 genome-wide, **p<0.01 chromosome-wide and *p<0.05 chromosome-wide significance level, #p<0.05 significance level of not annotated SNPs; ^8^: proportion of the explained variation (Var, %).

No QTL was identified for androstenone in data set A. In subset B1 and B2 two and five SNPs, respectively, were found to be associated with androstenone. The boar taint component skatole was characterized by significant associations located on *sus scrofa* chromosome (SSC) 10, 12 and 17 in the whole data set. The significant association on SSC17 was also detected in cross 1. Subset B2 revealed four associations for skatole on chromosome 7, 10, 16 and one SNP was not annotated. For the trait indole seven SNPs were significant in the whole population. One region on chromosome 14, where four SNPs were located, was also detected in subset B2, but not in subset B1. In subset B1 one region on SSC4 containing two SNPs was identified. In the same population the SNP on SSC1 affecting skatole was also associated with indole. Furthermore, this association explained 7.24% and 6.74% of the phenotypic variation of skatole and indole, respectively. For most of the other detected associations the proportion of the phenotypic variation explained ranged from 2.18% to 6.64%.

For testicular traits 31 significant associations were identified in this study (Table 2, 3 and 4). Twenty six SNP markers were located on autosomes and one was not yet mapped. The proportion of the explained phenotypic variance ranged 2.54 to 7.75%. The highest number of significant associations (22) was identified investigating the whole dataset. On SSC5 seven SNPs were identified affecting testicular weight. The substitution effect was between −10.12 (±2.76) g and −6.81 (±1.71) g. In subset B1 only two SNPs located on SSC12 and 18 revealed significant effects on GSI and testicular width, respectively. In the subset B2 seven significant associations affecting testicular traits were identified. Three SNPs are not mapped to the current reference porcine genome. Similar to boar taint traits, no common association was detected across all analyses for testicular traits.

### Principal component analysis

In order to investigate the joint genetic background of the androstenone synthesis and morphologic characteristics of the testes, PC comprising androstenone and GSI were calculated. The first PC explained 69.7 % of the total variance and had a negative canonical correlation (rc) to androstenone and GSI. The second PC explained 30.3 % of the variance and revealed a positive rc of 0.55 to androstenone and a negative rc of −0.55 to GSI.

In addition, the metabolism of boar taint components was analyzed applying principal component techniques to androstenone, skatole and indole. The first calculated PC1 had a strong negative rc to all boar taint components and explained 63.7% of their total variance (Table 5 and 6). All elements of the eigenvector had a negative sign and ranged from −0.49 to −0.64, which indicates a similar importance of all boar taint components. The second PC2 explained 24.9 % of the variance and rc had a positive sign for androstenone and negative for skatole and indole. PC2 was dominated by androstenone with an eigenvalue element of 0.84. PC3 explains the remaining 11.4 % of the total variance, and skatole and indole was more important than androstenone.

**Table 5:** **Loading and proportion of the variance explained by each principal component**

	**Eigenvectors** ^**2**^			**Total**
**Traits** ^**1**^	**PC**_**1L**_	**PC**_**2L**_	**PC**_**3L**_	
lnAND	−0.4875	0.8374	0.2471	
lnSKA	−0.5903	−0.5246	0.6135	
lnIND	−0.6433	−0.1532	−0.7501	
Eigenvalues	1.9099	0.7490	0.3412	3.0000
Proportion of total eigenvalues	0.6366	0.2497	0.1137	1.0000

^1^androstenone, log-transformed (lnAND); skatole, log-transformed (lnSKA); indole, log-transformed (lnIND); ^2^principal component (PC) 1 : 3 from the analysis of androstenone, skatole and indole (PC_1L_, PC_2L_, PC_3L_).

**Table 6:** **Canonical correlations of boar taint traits and principal components**

	**Trait** ^**1**^ **(ng/g fat, log transformed)**		
**Canonical variable** ^**2**^	**lnAND**	**lnSKA**	**lnIND**
PC_1L_	−0.67	−0.82	−0.89
PC_2L_	0.72	−0.45	−0.13
PC_3L_	0.14	0.36	−0.44

^1^androstenone, log-transformed (lnAND); skatole, log-transformed (lnSKA); indole, log-transformed (lnIND); ^2^principal component (PC) 1 : 3 from the analysis of androstenone, skatole and indole (PC_1L_, PC_2L_, PC_3L_).

GWAS were applied for the five determined PCs, which integrate androstenone and GSI (2) or all three boar taint components (3). Overall, 18 associations using the data set A (Additional file 1: Table S3) have been detected, by using these PCs. Moreover, analyzing the subsets B1 and B2, resulted in six and nine significant associations (Additional file 1: Table S4 and S5).

By comparing the results of the single trait GWAS with the PC analysis, 17 additional associations were detected of which six were located close to previous identified associations.

### Haplotype analysis

**Figure 3:**
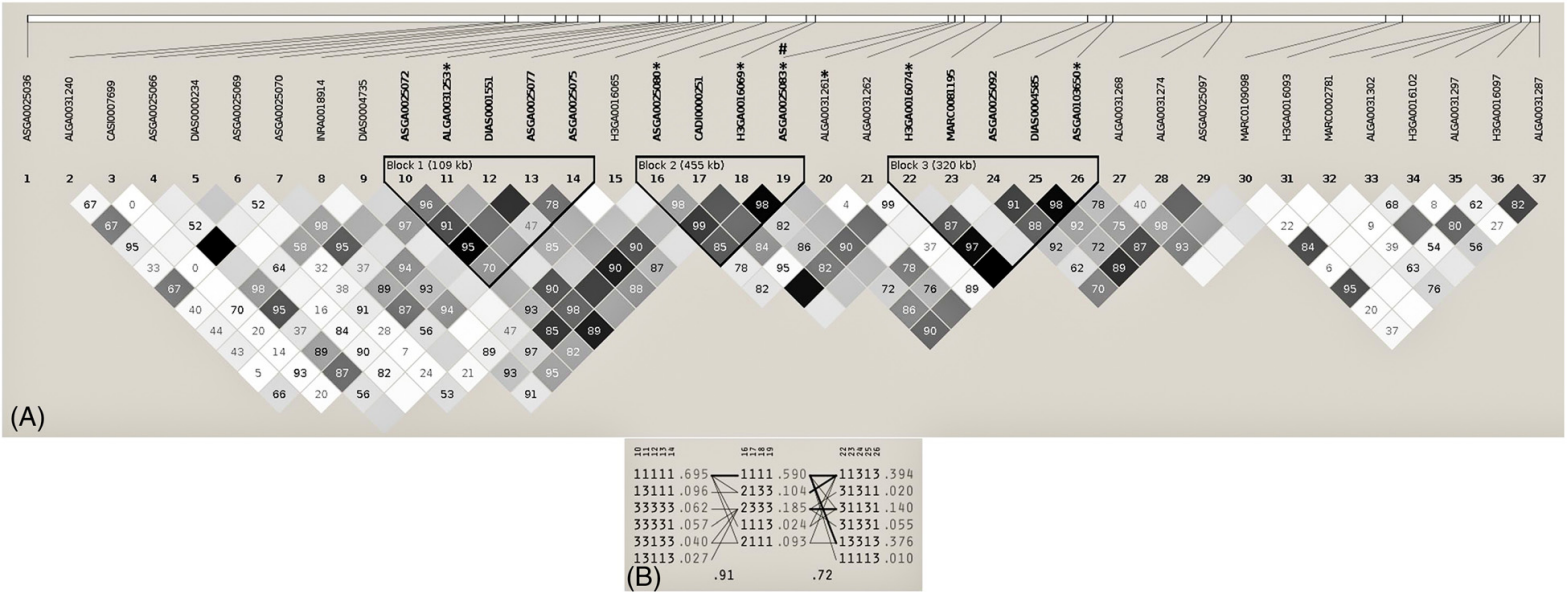
**Linkage disequilibrium plot for the region between 21.3 Mb and 25.1 Mb on SSC5 in subset B2. (A)** All significant SNPs for testicular weight (marked with *) (p ≤ 0.05 after Bonferroni correction) investigating the data set A (N = 598) and significant SNPs for gonadosomatic index (marked with #) (p ≤ 0.05 after Bonferroni correction) in subset B2, and all intervening SNPs for the boars of subset B2 (N = 356) are displayed. **(B)** Haplotypes for the 28 SNPs are shown. Each line represents a haplotype and the frequency of the haplotype in the population is given at each end of a line. Haplotypes below a frequency of 1.5% were excluded.

**Figure 4:**
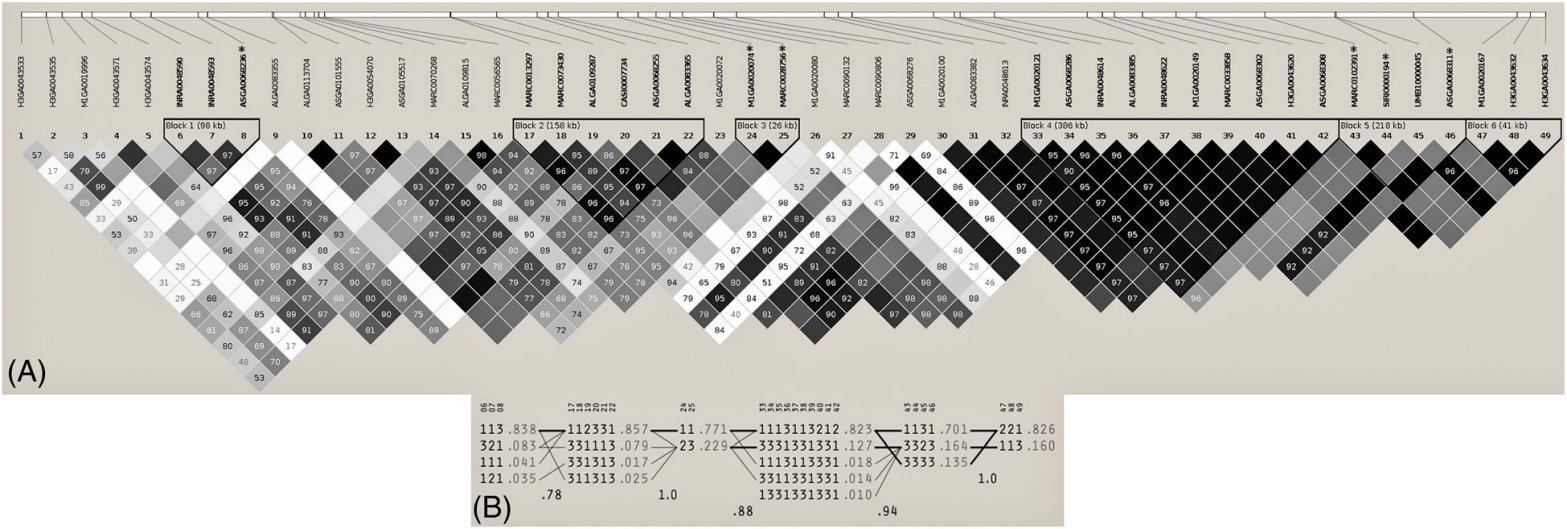
**Linkage disequilibrium plot for the region between 151.5 Mb and 154.0 Mb on SSC14 in subset B2. (A)** All significant SNPs for indole (marked with *) (p ≤ 0.05 after Bonferroni correction) and all intervening SNPs for the boars of subset B2 (N = 356, blue boxes) are displayed. **(B)** Haplotypes for the 28 SNPs are shown. Each line represents a haplotype and the frequency of the haplotype in the population is given at each end of a line. Haplotypes below a frequency of 1.5% were excluded.

Haplotype analyses were performed on basis of the whole data set as well as in the subsets B1 and B2. Haplotype blocks were defined, following the criteria of Gabriel *et al.* [45]. Distinct linkage disequilibrium (LD) and haplotype blocks were detected on SSC5 and SSC14 only in subset B2. The corresponding haplotype frequencies and the LD statistics (D’) are given in Figure 3 and Figure 4.

For the traits testes weight and GSI a significant region on SSC5 was detected. Within this region, three haplotype blocks were identified. The blocks were characterized by four or five SNPs and the D’ values between the neighboring blocks were 0.91 and 0.72 (Figure 3). Within this block seven SNPs had a significant association to testes weight and GSI.

On SSC14, a QTL for indole was identified. Haplotype analysis revealed six different blocks. The largest block comprised 10 SNPs, but none of these SNP exceeded the 5% significance threshold. The remaining five blocks contained less SNPs, but at least one of these showed a significant association to indole. The LD between neighbored haplotype blocks on SSC14 ranged between 0.78 and 1 (Figure 4).

In order to clarify the role of significant unmapped markers, LD between significant mapped and unmapped SNPs was estimated (Additional file 1: Figure S4). Out of the eight unmapped associations, four SNP markers showed high LD to trait-specific significant makers with known chromosomal position. The LD expressed as r^2^ ranged between 0.69 and 0.99. From this follows that both, unmapped and mapped SNPs within a haplotype block point to the same QTL. The role of the remaining four unmapped markers needs further investigation.

## Discussion

### The observed phenotypes and population structure

The raw means of recorded boar taint components revealed distinct differences between the crosses (Figure 1). Boars belonging to the group cross I had a higher level and variation for androstenone compared to the crosses II and III. The deviations in the mean between geographical regions can be explained by the genetic background of the F1 dams, obtained from three different breeding organizations. Moreover, no individual knowledge about the familiar relationships of the mated F1 sows was available. It can be speculated that the genetic heterogeneity of the F1 sows is reasonable for the high variation within cross I group. There are indications about the existing of an extend breeding stock exchange in the recent years of animals of the breed Pietrain [46].

Furthermore, it can be assumed, that the different F1 sow populations lead to population stratification. This was observed by visual inspection of Figure 2. In order to investigate genetically homogeneous populations, the data set was split according to the genetic background of the dams, accepting that the power decreases because of reduced number of animals. Similar observations regarding the genetic variation of dam lines have been described by Bergfelder-Drüing et al. [47].

In order to control the familiar half sib structure of the boars the GRAMMAR approach, as implemented in the GenABEL package [36], was applied. GRAMMAR is a widely used methodology for GWAS analysis and has been performed for different traits and species [14,48,49]. In this analysis the genomic relationship among all individuals is considered and allows controlling inflation due to stratification. Such a pedigree-based method could exploit inter-family variation in addition to intra-family variation, and could rapidly analyze hundreds of thousands of markers [39,40]. Additionally, the genomic control was used to account for spurious association due to population stratification [50], because the boars investigated in this study belong to a composite line of three different breeds. In this study no deviation from the chi-square distribution under the null-hypothesis of no association was observed. Trait and population specific Q-Q plots (Additional file 1: Figure S1-S3) contain regression lines which were calculated by a linear regression of expected (independent variable) and on observed test statistic (dependent variable). The slopes of these lines correspond to the calculated inflation factor, which is 1 in all analyses performed. This shows that possibly existing stratifications of the populations do not adversely affect the validity of corresponding GWAS analysis.

### Identified QTL and associations for individual traits of boar taint components

Comparing the number of detected associations and the resulting proportion of the explained phenotypic variance revealed large differences between the data sets (Table 7). Table 7 contains a summary of all identified QTL, where a QTL was defined by using a 1 Mb interval around the significant SNPs.

**Table 7:** **Summary of association analysis**

**Trait**	**Data set**	**Number of QTL**	**Expl. variation** ^**1**^
Androstenone	A	0	-
	B1	4	23.21%
	B2	2	7.85%
Skatole	A	3	7.08%
	B1	3	19.10%
	B2	4	17.92%
Indole	A	4	16.52%
	B1	2	13.18%
	B2	3	13.40%
testes length	A	5	14.34%
	B1	0	-
	B2	1	4.71%
testes width	A	1	2.54%
	B1	1	7.75%
	B2	1	3.78%
testes weight	A	5	17.54%
	B1	0	-
	B2	2	6.39%
GSI^2^	A	2	5.70%
	B1	1	7.57%
	B2	3	11.79%

^1^QTL was defined by using a 1 Mb interval around the significant SNPs; ^2^GSI: gonadosomatic index.

For instance, for androstenone no QTL was found analyzing the complete data set, whereas five and two significant SNPs were identified in subset B1 and B2, respectively. Summarizing the proportion of the explained phenotypic variance of the identified QTL, in subset B1 23.21% of the variation of androstenone was explained by four QTL (Table 7). In contrast, in subset B2 only 2 QTL for androstenone were identified explaining 7.85% of the phenotypic variance. Furthermore, no QTL was found across the two sub sets, indicating that androstenone is influenced by different alleles in the populations. Because there are no overlapping QTL, it is questionable if combination of genetically divergent populations is promising for detecting relevant QTL or improving the accuracy of genomic selection. Similar findings have been reported by Bergfelder-Drüing et al. [47] who investigated litter size in two maternal pig breeds.

**Figure 5:**
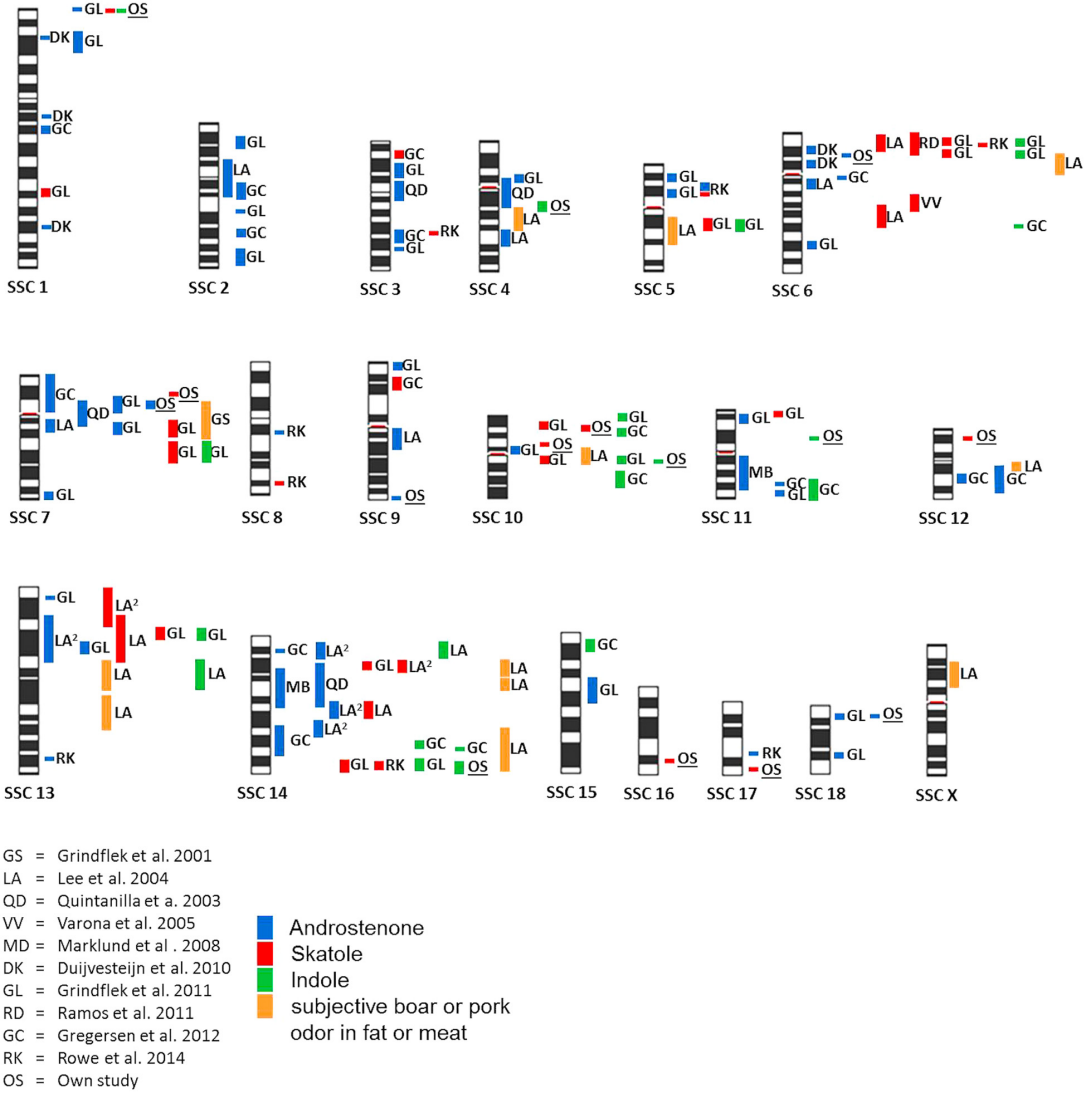
**Overview about identified QTL for boar taint components.** The plot visualized all identified QTL for boar taint components (androstenone, skatole and indole) and for subjective identified pork or boar odor. (LA^2^: The boar taint components were evaluated by a sensorial panel).

So far several studies have been conducted to investigate the association of boar taint or pork odor in meat [13-17,51-55] (Figure 5). The comparison of our results revealed that except of eight associations most of the regions were in accordance with reported QTL regions characterizing the development of pork odor. In general, comparing all identified QTL across the genome affecting androstenone, it can be seen that this trait is characterized by a polygenic inheritance (Figure 5). Although, most of the associated SNPs might be undetected because of small effects, this polygenic inheritance combined with high heritability is promising for genomic selection.

Additionally, eight SNPs detected here have not been annotated yet. These mutations explained up to 6% of the phenotypic proportion and might therefore be important for genomic selection and further functional studies.

Skatole and indole are influenced mainly by five chromosomal regions on SSC6, 7, 10, 13 and 14 described in five different studies [15-17,52]. The QTL on SSC 7, 10 and 14 were in accordance to our findings. In addition, significant associations were identified on SSC 1, 12, 16 and 17.

On SSC1 an association for skatole and indole was found in subset B1. Grindflek *et al.* [16] reported on this chromosome QTLs for androstenone and skatole, but not for indole. This described QTL for skatole [16] was not in accordance with our results.

On SSC4 a QTL for indole was identified in subset B1. In the same region Quintanilla *et al.* [55] and Lee *et al.* [17] detected a QTL for androstenone and boar odor, which was subjectively scored by test persons. In this region the genes TTPA (*tocopherol (alpha) transfer protein*) is localized, but until now the role of this gene has not been investigated in pigs. In humans, TTPA plays a role in vitamin E supply but is also involved in lipid and steroid binding and transport processes [56]. According to the lipophilic properties of indole and vitamin E it can be assumed that common metabolic processes in liver and transport mechanisms in fat are shared.

Analysis of subset B2 revealed one significant association for androstenone on SSC6. The identified mutation here was located between QTLs for androstenone described in the studies of Duijvesteijn *et al.* [51] and Grindflek *et al.* [16].

On SSC7 two significant associations were identified for androstenone. In this region several authors have reported QTLs related to androstenone [15,16,19]. The detected SNPs here were located in non coding regions, but surrounded by numerous genes.

The largest QTL region was detected on SSC14 associated with indole. In addition, six different haplotypes were detected which could be assigned to the particular associations. This was in accordance to earlier QTL studies, which reported associations for all three boar taint components and subjective odor scores [15-17]. Gregersen *et al.* [15] identified this QTL associated with indole in the breeds Landrace and Yorkshire. Within this QTL the functional gene CYP2E1 (*cytochrome P450, family 2, subfamily E, polypeptide 1*) has been described to be associated with boar taint. This gene is involved in the metabolism phase 1 of skatole in pigs (reviewed by [6,57]). In the present study two SNP markers located around CYP2E1 were significant associated with indole. The SNP UMB10000045, located in an intronic region of CYP2E1, was not significantly associated here. Similar observations have been described by Gregersen *et al.* [15]. In contrast, a study of Mörlein *et al.* [58] revealed that a mutation in the promotor of CYP2E1 influenced the levels of skatole and indole. It can be assumed that the associated markers are in linkage disequilibrium with the mutation in the promotor of CYP2E1 but may be not with the SNP located in the intron.

### Identified QTL and associations for individual traits of testicular characteristics

Until now, three studies identified QTLs influencing testicular traits in boars [59-61]. In our study a QTL region on SSC5 was detected for testicular length, testicular weight and GSI analyzing the data set A. QTLs for testes weight have been reported by Ren *et al.* [60] in a White Duroc × Erhualian resource population for testicular weight. These were not in accordance to our findings. In the region identified here, Cassady *et al.* [62] reported a QTL affecting the number of stillborn piglets.

The identified associations located on SSC4, 11, 12, 15 and 18 affecting testes traits have not reported or discussed so far.

### The investigation of pleiotropy using principal components

Pleiotropy is responsible for stable phenotypic and genetic correlations that can be observed between complex traits where a locus affects different traits [28]. This might be helpful for genomic selection when negatively correlated traits are processed. Furthermore, Solovieff et al. [63] reviewed several cross phenotype associations related to human diseases and showed that the underlying traits share some common genetic pathways that underscore the relevance of pleiotropy in complex traits.

In the present population estimates of genetic correlations between androstenone and skatole/indole ranged from 0.33 to 0.71 [29]. In this study androstenone, skatole and indole were investigated together using principal component techniques in order to identify QTL influencing the metabolism of all three boar taint components. In addition, androstenone was analyzed jointly with GSI in order to detect chromosomal regions involved in the synthesis of androstenone that is synthesized in the leydig cells of the testes [25]. Frieden et al. [29] estimated genetic correlations between 0.45 and 0.54 for androstenone and testes morphological parameters. Therefore pleiotropic effects can be expected.

In order to condensate the numbers of traits to one or two important variables principal component techniques were applied here. Such a multitrait analysis might be particularly beneficial in a situation, where the effect of a pleiotropic locus is too small to be detected by single-trait analyses only [64]. Several authors reported that the analysis of PCs were generally more powerful and accurate than single trait analyses [64-66], although a physiological interpretation is difficult, especially when a locus has an antagonistic effect on two traits.

The first, most important PC, which comprises all boar taint components with equal negative signs and similar weightings, supports the findings of and Doran *et al.* [10] who described that androstenone inhibits the degradation of skatole. However, nonlinear interactions between the boar taint components exists and have been reported [67]. Although biological unknown, these interaction might be statistically covered by the second and third PC, which includes androstenone and skatole/indole with adversative signs.

In a similar way, the results of the analysis of androstenone and GSI can be interpreted. The first PC explained 70% of the phenotypic variation with equal signs of the underlying variables. This is the statistical expression of the biological conjunction of androstenone level and morphological features of the testes which are the place of the synthesis. The remaining 30% of the variation is explained by the second PC, which includes androstenone and GSI with adversative signs and could be regarded as an antagonistic relationship between these two traits.

These interpretations of the PC demonstrate the usefulness of multidimensional analysis. However, according to Liu et al. [68] and Mahler *et al.* [69] only the first PC that explaining the majority of variation is necessary to analyze. In contrast, Olson *et al.* [70] showed that the highest phenotypic proportion explained by a genetic marker is not necessarily identified by the first PC. They argued that for a complex trait such a phenomena is not unexpected, because many factors influence a phenotype and the contribution of any individual gene to overall phenotypic variation might be small [70]. The importance of the second and following PCs has been investigated by Aschard *et al.* [71]. These authors showed that especially the PCs explaining a small amount of phenotypic variance can harbor a substantial part of the total genetic association and seemed to be very powerful, when QTL effects are opposite to positively correlated traits [71].

Based on the analyses of the PCs, GWAS revealed 31 significant associations from which 18 were not detected so far by means of single boar taint or testes phenotypes. Significant associations for PC, which comprises the boar taint components, were mainly identified on SSC 14 (4) and 16 (5). The QTLs on SSC16 have not been reported so far. Analyzing the PCs containing androstenone and GSI as underlying variables allowed to identify a QTL on SSC8. Until now no QTL for androstenone or testicular traits has been reported on SSC8, but several studies detected QTLs and associations in this chromosomal region affecting ovulation rate and age at puberty of sows [72,73].

All significant QTL found in this dataset were not directly linked to known functional candidate genes for boar taint or male fertility. A potential biological interpretation can be given for the significant marker ASGA0084322, which explained 8.44% of the phenotypic variation. This SNP, is localized in an intron region of a gene that has not been characterized yet, but upstream (~9 Mb) of this QTL the gene *α-Mannosidase 2, B3* (MAN2B2) has been mapped and identified mutations were significantly associated with ovulation rate [74]. Downstream (0.3 Mb) of the identified QTL, the gene *ligand dependent nuclear receptor corepressor-like* (LCORL) is localized that is associated with height growth and birth weight in human [75,76]. It has been shown by Salih *et al.* [77] that genes are clustered in regions that contribute to a particular quantitative trait. Against this background, it can be hypothesized, that the identified QTL affecting testicular morphology and the concentration of androstenone in backfat also influence reproduction performance in sows as well. In such a situation, selection strategies against boar taint might lead to delayed puberty and/or critical changes in ovulations rates, which underline the antagonistic expectation of the relationship between boar taint and fertility. In order to clarify this hypothesis and the underlying biological background of feasible pleiotropic effects on boar taint and reproduction traits, further studies with more flanking SNP in the region of marker ASGA0084322 are needed.

Different definitions of cross phenotype associations have been extensively reviewed and discussed for human complex traits by Solovieff *et al.* [63]. Beside biological pleiotropy (one gene/genetic variant affects two different traits) these authors described mediated pleiotropy (one phenotype is casual for a second phenotype and a genetic variant is associated with the first phenotype) and spurious pleiotropy (a falsely observed pleitropy or a high linkage). Regarding the applied method a genetical interpretation of the identified association is impossible, because it is unclear if there is “real” pleiotropy or a high linkage between two regions. In general, it should be considered that this analysis is a first step to identify pleitropic regions, which would have to be further examined with more specific models or molecular experiments [65].

## Conclusions

Based on Pietrain sired cross bred boars, the mayor objective of our study was to identify QTL for boar taint components and to detect pleiotropy among boar taint and testes traits. The results suggest that there are genetic differences between the crossbred lines leading to specific significant associations, which are probably induced by differences in the genetic background of the dam lines. The high number of identified QTLs and associations revealed that all three boar taint components are influenced by a large number of loci, which had an additive effect on all traits. Because of this polygenetic perception, genomic selection strategies are promising tools to improve boar taint components. Applying principal component techniques on the boar taint components allowed to clarify associations between boar taint components and can help to encode possible pleiotropic patterns. In this context it should be also possible to detect suspected antagonistic relationship between boar taint and testicular morphology. This is demonstrated by our GWAS, which was based on PC comprising boar taint components and morphological features of the testes. The identified regions are of special interest for genomic selection, because it should be avoided that selection response against boar taint lead to a decrease in reproduction performance.

## Additional file

Additional file 1:
**Table S1a.** Genetic (r_g_) and phenotypic correlations (r_p_) between boar taint components and corresponding heritability (h^2^) (± standard error) (Frieden et al. 2014); **Table S1b.** Genetic (r_g_) and phenotypic correlations (r_p_) between boar taint components and testes morphology and corresponding heritability (h^2^) (± standard error) (Frieden et al. 2014); **Table S2.** Genome-wide and chromosome-wide critical values for the significance levels of the empirical p-value after Bonferroni correction; **Table S3.** Chromosome-wide significant associations identified in all 598 boars for calculated principal components characterizing the biosynthesis and metabolism of androstenone; **Table S4.** Chromosome-wide significant associations identified in subset B1 for calculated principal components characterizing the biosynthesis and metabolism of androstenone; **Table S5.** Chromosome-wide significant associations identified in subset B2 for calculated principal components characterizing the biosynthesis and metabolism of androstenone; **Figure S1.** QQ-plots of GWAS for boar taint components; **Figure S2.** QQ-plots of GWAS for testes morphological parameters; **Figure S3.** QQ-plots of GWAS for principal components; **Figure S4.** Linkage disequilibrium plots for all significant SNPs.
