# Supplementary material for: Genome-wide association analyses for boar taint components and testicular traits revealed regions having pleiotropic effects
Source: BMC Genet. 2015 Apr 9;16:36. doi: 10.1186/s12863-015-0194-z (PMC4429935; doi:10.1186/s12863-015-0194-z)
Supplement: Additional file 1: — Table S1a. Genetic (rg) and phenotypic correlations (rp) between boar taint components and corresponding heritability (h2) (± standard error) (Frieden et al. 2014); Table S1b. Genetic (rg) and phenotypic correlations (rp) between boar taint components and testes morphology and corresponding heritability (h2) (± standard error) (Frieden et al. 2014); Table S2. Genome-wide and chromosome-wide critical values for the significance levels of the empirical p-value after Bonferroni correction; Table S3. Chromosome-wide significant associations identified in all 598 boars for calculated principal components characterizing the biosynthesis and metabolism of androstenone; Table S4. Chromosome-wide significant associations identified in subset B1 for calculated principal components characterizing the biosynthesis and metabolism of androstenone; Table S5. Chromosome-wide significant associations identified in subset B2 for calculated principal components characterizing the biosynthesis and metabolism of androstenone; Figure S1. QQ-plots of GWAS for boar taint components; Figure S2. QQ-plots of GWAS for testes morphological parameters; Figure S3. QQ-plots of GWAS for principal components; Figure S4. Linkage disequilibrium plots for all significant SNPs. [file 12863_2015_194_MOESM1_ESM.pdf]

Supplementary

Table S1a - Genetic (r_g) and phenotypic correlations (r_p) between boar taint components and corresponding heritability (h^2) (± standard error) (Frieden et al. 2014)

	Skatole (ln)		Indole (ln)		
	r_g	r_p	r_g	r_p	h^2
Androstenone (ln)	0,33 ± 0,12	0,29	0,68 ± 0,11	,39	0,60 ± 0,11
Skatole (ln)			0,71 ± 0,08	,62	0,43 ± 0,06
Indole (ln)					0,32 ± 0,08

ln: log transformed

Table S1b - Genetic (r_g) and phenotypic correlations (r_p) between boar taint components and testes morphology and corresponding heritability (h^2) (± standard error) (Frieden et al. 2014)

	Androstenone (ln)		Skatole (ln)		
	r_g	r_p	r_g	r_p	h^2
Testes width	0,54 ± 0,22	0,21	0,12 ± 0,24	0,10	0,11 ± 0,06
Testes weight	0,45 ± 0,15	0,34	0,26 ± 0,17	0,15	0,38 ± 0,07
Testes length	0,53 ± 0,28	0,18	0,30 ± 0,25	0,09	0,07 ± 0,05

ln: log transformed

Frieden L, Große-Brinkhaus C, Neuhoff C, Schellander K, Looft C, Tholen E: **Möglichkeiten zur Reduktion von geschlechtsbedingten Geruchsabweichungen am Schlachtkörper von männlichen, unkastrierten Mastschweinen - Teil 2: Genetische Fundierung des Merkmals Ebergeruch und genetische Beziehungen zu paternalen und maternalen Reproduktionsleistungen. Reduktion Ebergeruch**. *Züchtungskunde* 2014:319-341.

Table S2 - Genome-wide and chromosome-wide critical values for the significance levels of the empirical p-value after Bonferroni correction

	entired data set			data set B1			data set B2		
Chromosome SSC	No. of SNP	0.05	0.01	No. of SNP	0.05	0.01	No. of SNP	0.05	0.01
1	5193	9.63E-06	1.93E-06	5500	9.09E-06	1.82E-06	5345	9.35E-06	1.87E-06
2	2842	1.76E-05	3.52E-06	2929	1.71E-05	3.41E-06	2961	1.69E-05	3.38E-06
3	2329	2.15E-05	4.29E-06	2499	2.00E-05	4.00E-06	2400	2.08E-05	4.17E-06
4	2996	1.67E-05	3.34E-06	3086	1.62E-05	3.24E-06	3108	1.61E-05	3.22E-06
5	1964	2.55E-05	5.09E-06	2083	2.40E-05	4.80E-06	2040	2.45E-05	4.90E-06
6	2541	1.97E-05	3.94E-06	2729	1.83E-05	3.66E-06	2721	1.84E-05	3.68E-06
7	2636	1.90E-05	3.79E-06	2724	1.84E-05	3.67E-06	2783	1.80E-05	3.59E-06
8	1716	2.91E-05	5.83E-06	1951	2.56E-05	5.13E-06	1899	2.63E-05	5.27E-06
9	2812	1.78E-05	3.56E-06	2912	1.72E-05	3.43E-06	2910	1.72E-05	3.44E-06
10	1509	3.31E-05	6.63E-06	1565	3.19E-05	6.39E-06	1573	3.18E-05	6.36E-06
11	1600	3.13E-05	6.25E-06	1665	3.00E-05	6.01E-06	1690	2.96E-05	5.92E-06
12	1293	3.87E-05	7.73E-06	1369	3.65E-05	7.30E-06	1360	3.68E-05	7.35E-06
13	3471	1.44E-05	2.88E-06	3598	1.39E-05	2.78E-06	3576	1.40E-05	2.80E-06
14	3242	1.54E-05	3.08E-06	3439	1.45E-05	2.91E-06	3325	1.50E-05	3.01E-06
15	2137	2.34E-05	4.68E-06	2311	2.16E-05	4.33E-06	2299	2.17E-05	4.35E-06
16	1570	3.18E-05	6.37E-06	1661	3.01E-05	6.02E-06	1658	3.02E-05	6.03E-06
17	1371	3.65E-05	7.29E-06	1449	3.45E-05	6.90E-06	1457	3.43E-05	6.86E-06
18	1014	4.93E-05	9.86E-06	1141	4.38E-05	8.76E-06	1067	4.69E-05	9.37E-06
NA	3835	1.30E-05	2.61E-06	4073	1.23E-05	2.46E-06	4037	1.24E-05	2.48E-06
Genome	42236	1.18E-06	2.37E-07	48684	1.03E-06	2.05E-07	48209	1.04E-06	2.07E-07

Table S3 - Chromosome-wide significant associations identified in all 598 boars for calculated principal components characterizing the biosynthesis and metabolism of androstenone

Trait[1]	SNP	SSC[2]	Pos[3]	Mut[4]	MAF[5]	Eff (se)[6]	Chi2	Emp. P-value[7]	Var[8]
PC_{1L}	ALGA0064494	12	7132733	G/A	0.23	-0.21(0.05)	14.60	1.84E-05*	2.59%
PC_{3L}	M1GA0020074	14	152454616	A/C	0.26	-0.11(0.03)	16.76	7.66E-06*	2.97%
PC_{3L}	MARC0028756	14	152480709	A/G	0.26	-0.11(0.03)	16.76	7.66E-06*	2.97%
PC_{3L}	SIRI0000194	14	153477507	A/G	0.32	-0.11(0.03)	17.15	6.02E-06*	3.03%
PC_{3L}	ASGA0068311	14	153593360	A/G	0.32	-0.10(0.03)	16.59	8.53E-06*	2.94%
PC_{2L}	ASGA0089810	16	75391932	A/C	0.42	0.08(0.02)	13.23	2.84E-05*	2.36%
PC_{2T}	ALGA0031253	5	23002609	A/G	0.33	0.10(0.02)	22.03	6.31E-08***	3.86%
PC_{2T}	ASGA0025080	5	23190382	A/C	0.40	0.08(0.02)	15.37	6.21E-06*	2.73%
PC_{2T}	CADI0000251	5	23291033	A/G	0.21	0.09(0.02)	14.02	1.59E-05*	2.49%
PC_{2T}	H3GA0016069	5	23313949	A/G	0.32	0.09(0.02)	19.62	3.30E-07***	3.46%
PC_{2T}	ASGA0025083	5	23646360	A/G	0.33	0.09(0.02)	21.09	1.20E-07***	3.71%
PC_{2T}	H3GA0016074	5	23737420	A/G	0.24	0.10(0.02)	17.79	1.17E-06***	3.14%
PC_{2T}	MARC0081195	5	23777569	A/G	0.40	-0.07(0.02)	14.01	1.60E-05**	2.49%
PC_{2T}	DIAS0004585	5	24041995	A/G	0.23	0.09(0.02)	15.94	4.19E-06**	2.83%
PC_{2T}	ASGA0103650	5	24057900	G/A	0.24	0.10(0.02)	17.77	1.18E-06***	3.14%
PC_{2T}	ALGA0031274	5	24330746	G/A	0.35	0.08(0.02)	14.00	1.62E-05*	2.49%
PC_{2T}	MARC0003381	NA	NA	A/G	0.44	0.09(0.02)	24.71	1.01E-08#	4.31%
PC_{2T}	ASGA0083183	NA	NA	A/G	0.24	0.10(0.02)	17.79	1.17E-06#	3.14%

[1] principal component (PC) 1 : 3 from the analysis of androstenone, skatole and indole (PC_{1L}, PC_{2L}, PC_{3L}) and androstenone and gonadosomatic index (GSI) (PC_{1T}, PC_{2T}); [2]: *sus scrofa* chromosome (SSC); [3]: position in Mb; [4]: mutation (MUT); [5]: minor allele frequency (MAF); [6]: substitution effect and standard error (se), [7]: empirical p-value and significant thresholds, Bonferroni corrected - *** p< 0.05 genome-wide, **p<0.01 chromosome-wide and *p<0.05 chromosome-wide; [8]: proportion of the explained variation (Var, %)

Table S4 - Chromosome-wide significant associations identified in subset B1 for calculated principal components characterizing the biosynthesis and metabolism of androstenone

Trait[1]	SNP	SSC[2]	Pos[3]	Mut[4]	MAF[5]	Eff (se)[6]	Chi2	Emp. P-value[7]	Var[8]
PC_{1L}	H3GA0000454	1	6764028	A/C	0.45	0.35(0.09)	16.84	7.58E-06*	7.99%
PC_{3L}	MARC0055531	17	62159866	A/G	0.29	-0.18(0.05)	15.87	1.64E-05*	7.56%
PC_{1T}	ASGA0084322	8	12390177	C/A	0.23	0.36(0.09)	17.89	5.46E-06**	8.44%
PC_{1T}	ALGA0108110	8	12407569	C/A	0.25	0.33(0.09)	15.20	2.78E-05*	7.27%
PC_{1T}	ALGA0066656	12	46290007	A/G	0.38	0.27(0.07)	15.25	2.70E-05*	7.29%
PC_{2T}	MARC0023053	10	70798390	C/A	0.10	-0.15(0.04)	14.64	1.54E-05*	7.02%

[1] principal component (PC) 1 : 3 from the analysis of androstenone, skatole and indole (PC_{1L}, PC_{2L}, PC_{3L}) and androstenone and gonadosomatic index (GSI) (PC_{1T}, PC_{2T}); [2]: *sus scrofa* chromosome (SSC); [3]: position in Mb; [4]: mutation (MUT); [5]: minor allele frequency (MAF); [6]: substitution effect and standard error (se), [7]: empirical p-value and significant thresholds, Bonferroni corrected - *** p< 0.05 genome-wide, **p<0.01 chromosome-wide and *p<0.05 chromosome-wide; [8]: proportion of the explained variation (Var, %)

Table S5 - Chromosome-wide significant associations identified in subset B2 for calculated principal components characterizing the biosynthesis and metabolism of androstenone

Trait[1]	SNP	SSC[2]	Pos[3]	Mut[4]	MAF[5]	Eff (se)[6]	Chi2	Emp. P-value[7]	Var[8]
PC_{1L}	ASGA0033595	7	52462626	A/C	0.08	-0.25(0.07)	14.15	1.74E-05*	3.87%
PC_{1L}	ASGA0074339	16	78560312	A/G	0.27	-0.15(0.04)	13.42	2.85E-05*	3.67%
PC_{1L}	ASGA0085909	NA	NA	A/G	0.47	0.15(0.04)	15.95	5.05E-06#	4.35%
PC_{2L}	H3GA0046827	16	62825399	C/A	0.44	-0.07(0.02)	13.58	1.85E-05*	3.72%
PC_{2L}	H3GA0046828	16	62872977	G/A	0.45	-0.07(0.02)	14.31	1.11E-05*	3.91%
PC_{2L}	ASGA0089810	16	75391932	A/C	0.42	0.08(0.02)	18.16	7.40E-07***	4.91%
PC_{2T}	ALGA0031253	5	23002609	A/G	0.30	0.10(0.03)	15.51	7.29E-06*	4.22%
PC_{2T}	ASGA0025083	5	23646360	A/G	0.31	0.10(0.03)	15.19	9.08E-06*	4.14%
PC_{2T}	MARC0003381	NA	NA	A/G	0.42	0.10(0.03)	19.92	3.73E-07#	5.36%

[1] principal component (PC) 1 : 3 from the analysis of androstenone, skatole and indole (PC_{1L}, PC_{2L}, PC_{3L}) and androstenone and gonadosomatic index (GSI) (PC_{1T}, PC_{2T}); [2]: *sus scrofa* chromosome (SSC); [3]: position in Mb; [4]: mutation (MUT); [5]: minor allele frequency (MAF); [6]: substitution effect and standard error (se), [7]: empirical p-value and significant thresholds, Bonferroni corrected - *** p< 0.05 genome-wide, **p<0.01 chromosome-wide and *p<0.05 chromosome-wide; [8]: proportion of the explained variation (Var, %)

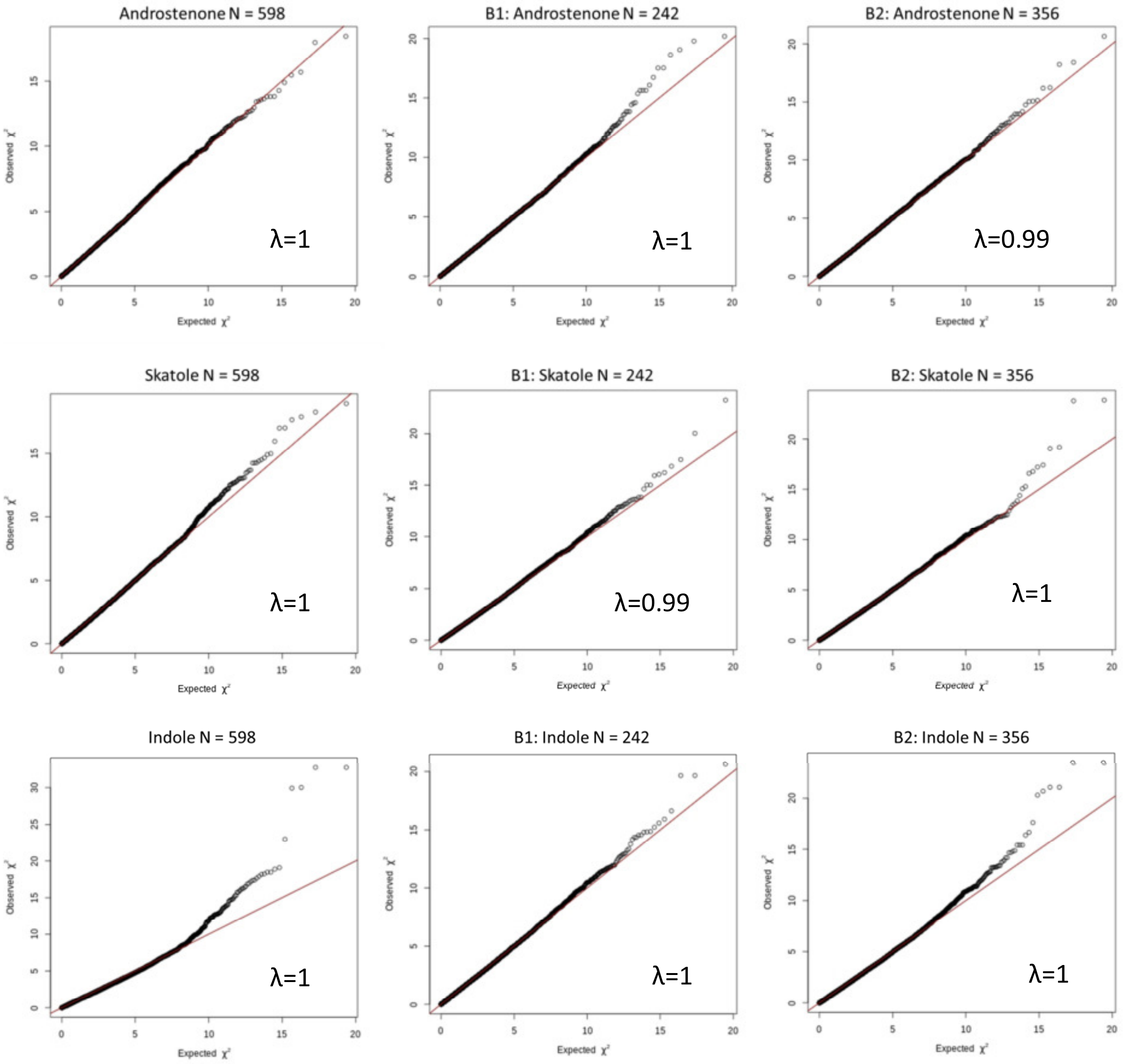

Figure S1 - QQ-plots of GWAS for boar taint components

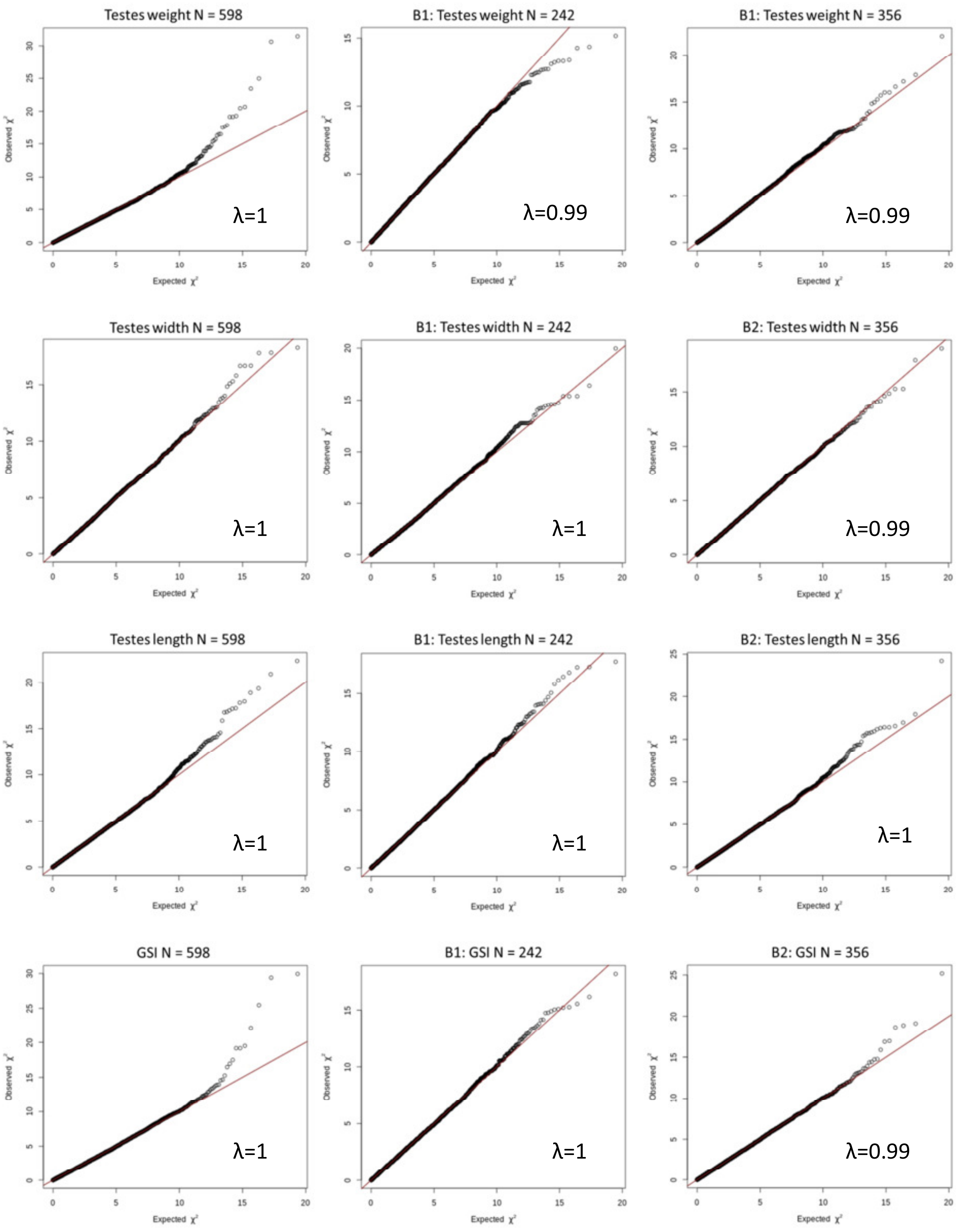

Figure S2 - QQ-plots of GWAS for testes morphological parameters

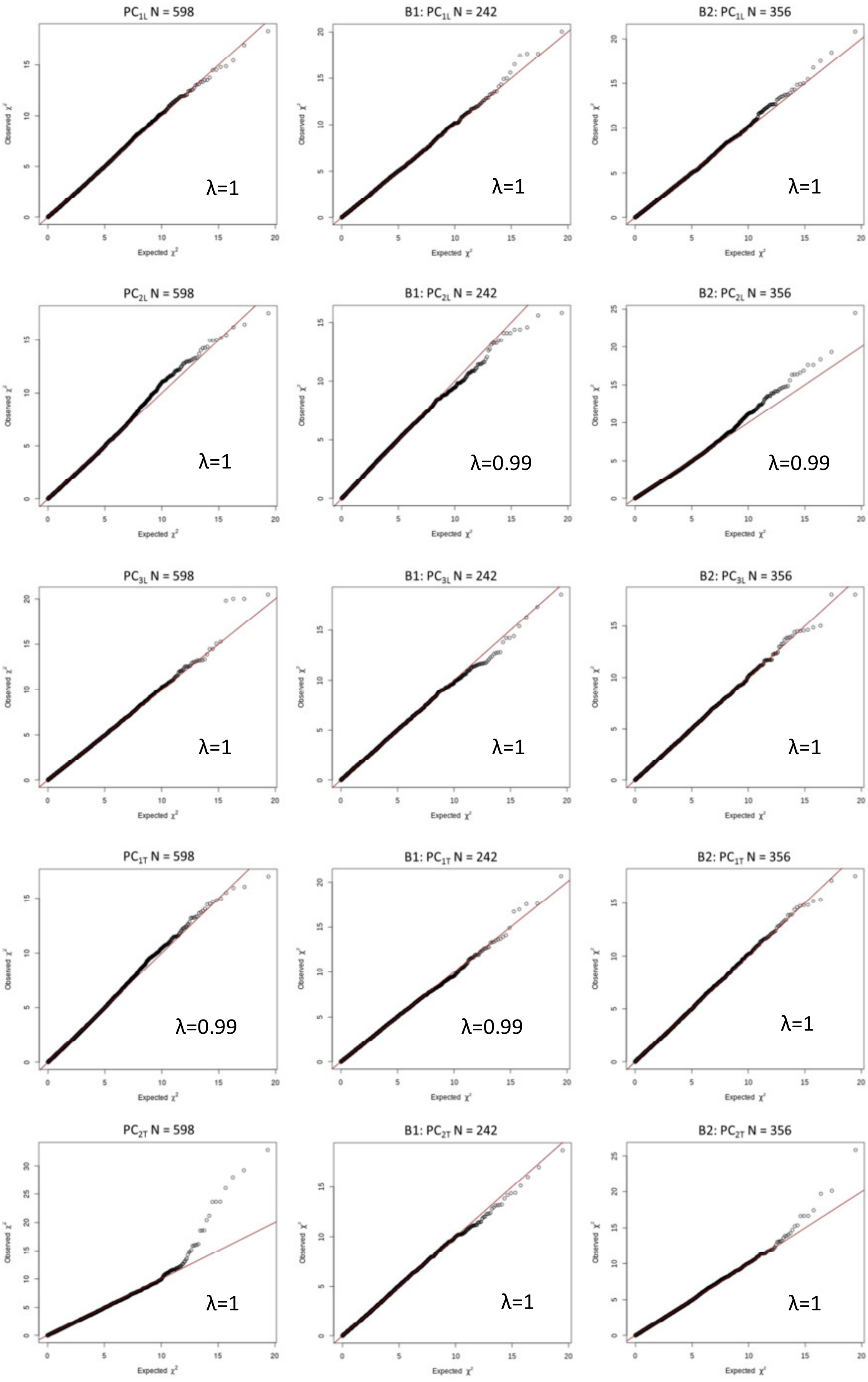

Figure S3 - QQ-plots of GWAS for principal components

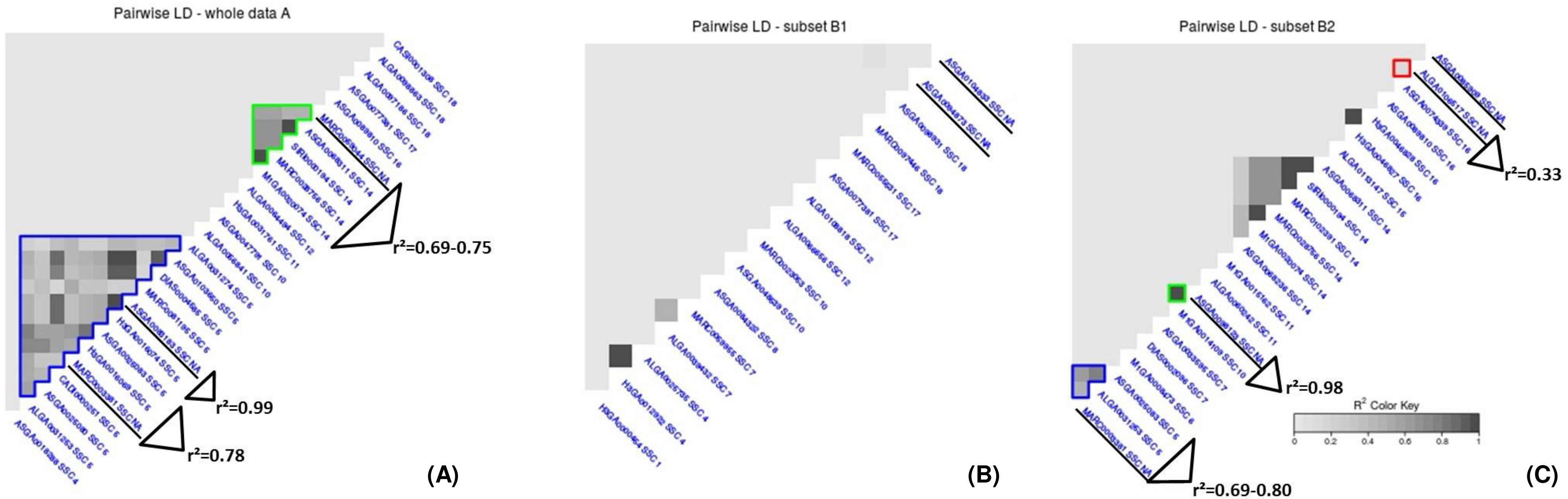

Figure S4 - Linkage disequilibrium plots for all significant SNPs

Linkage disequilibrium (LD) plots for SNP-markers significantly associated for boar taint traits, testes morphological parameters and principal components. The marker name is complemented by the porcine chromosome number (SSC) they are located on. SNPs that are unmapped so far are underlined and marked with “SSC NA”. In addition, these unmapped markers were inserted between mapped SNPs based on r^2 values. r^2 values between unmapped and mapped makers is given at the top of the triangles. **(A)** Linkage disequilibrium (LD) plot all significant SNPs identified for data set A, N = 598; **(B)** LD plot all significant SNPs identified for subset B1, N = 242; **(C)** LD plot all significant SNPs identified for subset B2, N = 356
